# The Story of the Insane from Year to Year

**Published:** 1904-08-27

**Authors:** 


### THE STORY OF THE INSANE FROM YEAR TO YEAR.

Sixteenth Annual Series.

### GLOUCESTER COUNTY ASYLUM.

At this asylum the numbers increased during the year from 1,017 to 1,087. There were 266 first admissions, 41 re-admissions, 81 recoveries, 12 discharged relieved, 22 not improved, and 118 died. The recoveries stand at 32 7 per cent. of the admissions, and the deaths at 11 21 per cent. of the average number resident. Of the deaths, 41 were due to cerebral and spinal diseases, 3 to pneumonia, and only 5 to consumption. Dr. Craddock, doubtless rightly, attributes this low mortality from phthisis, which is 4 per cent. of the total death-rate, to efficient sanitation and ventilation. And certainly it is wonderful what can be done to mitigate this scourge of phthisis even in the oldest of our asylums, by insisting on the patients spending much of the day out of doors, and sleeping by night in cross-ventilated rooms, especially if the wards be free from that poisonous artifically-heated air. That other asylum scourge, enteritis, has not yet been banished from the Gloucester asylums, for we notice that it killed, or helped to kill, six patients. Various moral causes alone, or in combination with others, accounted for the insanity in 82 of the year's admissions, and among the physical causes heredity, previous attacks, and intemperance in drink are the most frequent. In his well-put and well-written report Dr. Craddock discusses the increase in the numbers of the insane in his own county, and he fears, not without cause, that the result of his investigations will be received with little satisfaction either by the committee or the ratepayers or the county at large. He shows that Gloucestershire had in its asylums at the end of the year 1881, 688 lunatics, at the end of 1891 it had 829, and at the end of 1901 it had 953; or, to put it another way, the first decade showed an annual increase of 14, and the second an annual increase of 12, and that, too, after the boundaries of the Borough of Bristol had been extended and had taken into its fold a considerable slice of the county. Dr. Craddock is one of the medical superintendents who always fearlessly expresses his opinion, and he states once more that the chief causes of this deplorable increase are heredity and drink. "Hitherto," he says, "the methods proposed for the prevention and cure of insanity are about as hopeful and rational as the voyage to Anticyra recommended by the Augustan Satirist." And he is right. Not all the hellebore which the Phocian city ever produced would have any effect unless the dose were increased far beyond what Dr. Craddock dared prescribe. The following instance may not adorn the tale, but it points the moral. "A man who had been an inmate here more than once, on the last occasion for some five or six years, unexpectedly began to improve, and at length was so much better that his relatives wished to give him a trial at home. I willingly assented. Within a few months he was engaged to be married, and actually was married, it will hardly be credited, to the daughter of a woman who has been here for years, and is never likely to be anywhere else. It is difficult to find words to denounce what I hold to be the wickedness, the criminal nature, of such a union; and prohibitory legal enactments with the most stringent sanctions, would be not only justifiable but urgently demanded." The Gloucester Asylum contains many private and out-county patients, and last year the profits amounted to £1,288. The committee very properly decided some time since not to pay income tax on these so-called profits. The weekly cost per head was almost 8s. 11d. Out-county patients are charged from 14s. to 16s. a week, and private patients 15s.

### GLASGOW DISTRICT ASYLUM AT GARTLOCH.

At the beginning of the year this asylum contained 587 patients, and at the end of the year the number had risen to 621. There were 197 first admissions, 73 readmissions, 114 recoveries, 37 discharged relieved, and 70 died. The total number under treatment was 872, and the average number resident was 613. The recoveries stand at 40 per cent. of the admissions and the deaths at 11·4 per cent. of the average number resident. Of the year's deaths 36 were caused by cerebral and spinal diseases, 5 by consumption, and 8 by other lung diseases. Alcoholic intemperance was the cause of the insanity in 69 of the admissions, in 35 additional cases it was combined with other causes, and hereditary influence accounts for 65. It is always difficult to obtain a complete family history in cases sent to our asylums, and Dr. Parker tells us that in only 129 of the 285 admissions was this history reasonably complete. The sanatorium for consumptives has been in use for some time, and it would seem as if it had already reduced the death rate from phthisis, for it was only 8·5 per cent. of the total death rate, whereas in the previous five years the average was over 20 per cent. The usual classes for instructing the attendants and nurses in their duties were conducted during the year, and seven of the staff obtained the certificate. Dr. Parker publishes a "Note on Increase of Lunacy," from which it appears that since the opening of the asylum at the end of the year 1896 there has been an increase in the Gartloch Asylum population of 120. Of course, this increase is not all caused by increase of insanity, but partly depends on the fact that nowadays cases are sent to asylums that formerly would have been kept at their own homes, or in the workhouse. From whatever cause or causes, there is an undeniable increase in the number of the registered insane, and Dr. Parker finishes his Note as follows: "As regards the prevention of insanity, much may be done in the future by the stamping out of tuberculosis, by enforcing a Contagious Diseases Act, by legislating to prevent the marriages of epileptics and other degenerates, and by a more thorough control of the drink traffic."

